# Notes and News

**Published:** 1889-07-06

**Authors:** 


### Notes and News.

Collected and Communicated.

After the terrible tales of asylums with which novels have made many of us familiar, it is amusing to learn that Daniel O'Neil has been feigning madness for twenty-two years in order to gain admittance to them. Those cruel police now see through his lunatic dodges, and refuse to hand him over to the tender mercies of asylum superintendents.

We have had much pleasure in forwarding to the Hon. A. Lyttleton the two dollars donation, which we have received from a Philadelphia correspondent, in aid of the Children's Country Holiday Fund. We cordially echo the wish of our correspondent that this gift may make the whole year seem happier to one child at least. This donation is gratifying evidence of the interest taken in all good works by philanthropists on both sides of the Atlantic. Such workers rightly regard England and America as one and the same so far as their undertakings and interests are concerned.

The dental department at Guy's is now in full working order, and the 5,000 dental patients who visit the hospital annually can be sure of prompt and efficient attention. A room has been built within the hospital grounds, amply lit by windows and top lights, and with space for eighteen operating chairs. Every modern appliance has been furnished, including saliva ejectors and electrodes, with a constant supply of electricity. In this room all dental operations will be performed, except the extraction of teeth. Two extraction-rooms have been set apart in the general out-patients' department, and an anæsthetist is in attendance every morning. The staff includes, besides dental surgeons for every day in the week, lecturers on all the special subjects included in the dental curriculum.

Lady Edward Cavendish opened the Derbyshire Convalescent Home, Matlock Bank, on a glorious June day. The secretary said that the home which Lady Edward Cavendish was to open that day was the outgrowth of a smaller institution established by Mr. Newton, at Mickleover, in the year 1873. That home was largely used by the late Miss Brumwell, the lady superintendent of the Derbyshire Nursing Association, for the benefit of her sick poor in Derby. It was mainly due to this lady's energetic spirit that a larger scheme was set on foot to meet the increasing demand for such an institution. The home was arranged for 36 patients, and if fully occupied at an average of three weeks for each would accommodate about 500 patients per annum. That would necessitate an annual income derivable from subscribers of £500, and already promises had been received representing £70 per annum. After the opening proceedings a fête was held in the grounds, at which a large number of persons were present.

Mr. J. Forrester Wood, M.R.C.S., L.R.C.P., has been elected to the post of house surgeon to the General Infirmary at Gloucester, in the room of Mr. F. H. Knaggs resigned.

A dance at the Portman Rooms, under the auspices of Mrs. John Churchill and Miss Maud Kennedy, in aid of the Homœopathic Convalescent Home, Eastbourne, has resulted in a cheque for £108 4s. 5d. for that institution.

The Lord Mayor has received from the Rev. Prebendary Forrest, D.D., a cheque for £1,208 15s., being the result of the collections at St. Jude's, South Kensington, of which he is vicar, on Hospital Sunday. This is the largest collection received for the fund since its inauguration seventeen years ago; it exceeds by £200 the collection at St. Michael's, Chester-square, where the amount has averaged £1,000 for the last seven years.

For some time back the Local Government Board has brought considerable pressure to bear on the guardians of the Hanwell schools, in order to secure the appointment of a specialist to treat the existing ophthalmia. The district authorities have at length made their selection, which has been formally approved by the Local Government Board. Mr. Sydney Stephenson, M.B. (Edin.), F.R.C.S., has been appointed for twelve months at a salary of £500. A few years since, in the Lambeth Schools at Norwood, that gentleman had charge of a similar epidemic, in the stamping out of which he was entirely successful. Readers may be reminded that "granular tids"—as this form of ophthalmia is called—is a highly contagious disease, very destructive of sight, and requiring months or sometimes even years of active treatment for its cure. At Hanwell there are some twelve hundred children, one third of whom have been found to require isolation and treatment. The managers have determined to erect temporary buildings to accommodate 400 ophthalmic cases; and it is proposed to add some "isolation schools," so that the children's education will not be neglected. Mr. Stephenson has already commenced work, and no one who is acquainted with this gentleman's skill and energy can for a moment doubt that his efforts at Hanwell will be attended by the same distinguished success as formerly at Norwood.

A writer in the *Evening Standard* has given an account of a leper hospital in the West Indies, and he points out how much better is the fate of the West Indian than of the Asiatic leper. There, in India, there is as yet no segregation, no enforced hospital treatment, and the lepers wander about, a curse to themselves and to all they approach. This is a description of the leper hospital in which the writer of the article worked: "There, stretched upon the snowy bed, or seated at its side wrapped from head to foot in a blanket, never moving or speaking, scarcely breathing, overcome with a nameless horror—the horror and terror of a great desolation—one shudderingly beholds the poor wretch upon whose quivering frame the fell disease has well nigh done its worst. And passing down the long corridor, or stooping over to wet some parched and dying lips with cool, sparkling water, one sees, with love and thankfulness, the French Roman Catholic Sisters of Mercy, who devote their lives to the alleviation of such miseries as those I have feebly endeavoured to describe, and who, with incredible self-sacrifice, with undreamed-of heroism, and with utter self-forgetfulness, have given up all that makes life worth having that, of their tenderness, they may soothe the last moments on earth of these 'smitten o God and accursed.'"

The following vacancies are announced this week, the amount of the salary in each case being given after the name of the institution :

**Resident Medical Officers.**

West London Hospital, Hammersmith. House Surgeon and House Physician.—Board, &c.
Carlisle Infirmary. House Surgeon.—£60, board, &c.
Carlisle Infirmary. Assistant House Surgeon. — £40, board, &c.
Bury Dispensary Hospital. Junior House Surgeon.—£60, board, &c.
City of London Hospital for the Diseases of the Chest, Victoria Park. House Physician.—Board, &c.
Rubery Hill Asylum. Clinical Assistant.—Board, &c.

**Non-Resident Medical Officers.**

St. George's Hospital. Assistant Physician.
Queen's Hospital, Birmingham. Honorary Physician and Honorary Obstetric Officer.
King's College, London. Professor of Dental Surgery.
Victoria University. Demonstrator of Physiology.—£120.
King's College, London. Surgical Registrar.
Frome Union. Medical Officer.—£77.

Lord Aberdare presided at the annual meeting of the Society for the Prevention of Cruelty to Animals, and stated that one of the little essayists, in order to prove that it was wrong to cut off the tails of horses and cats, had quoted the scriptural injunction, "What God has joined together let no man put asunder.

The sale of work by the inmates of the Royal Hospital for Incurables, held at Putney last week, brought in over £400. The weather was delicious, and on two days the Police Band was in attendance. On Thursday the visitors were not so numerous as could have been desired, but the Windsor show doubtless carried away a good many. The Viscount and Viscountess of Portman opened the sale, and purchased from many of the stalls.

The *Chemist and Druggist* is giving a series of articles on London hospitals, chiefly from the dispensary point of view. On May 18 the paper dwelt with St. Bartholomew's Hospital, where the dispensers have few spare minutes during the nine hours. As this hospital has a pharmacopœia of its own (it was first published in 1718), many of the mixtures, lotions, ointments, pills, etc., are kept ready prepared, and a complete set of printed labels facilitates labour. The greatest caution is taken to prevent accidents with poisons, and in the hospital itself all potent preparations are kept in triangular-shaped bottles or pots, specially made.

The Children's Country Holidays Fund recently held a conference of its town and country workers at the Earl of Aberdeen's. Lord Kilmarnock was in the chair. He is by no means a merely ornamental chairman since in addition to his not very difficult duties as one of the trustees he is an active member of the executive committee and a local treasurer and secretary. The meeting was intended to give the country correspondents an opportunity of expressing their views and talking over their difficulties face to face with their town colleagues. The room was very full, and one or two vexed questions were well ventilated, but too few of the country people were heard, and no one brought forward grievances. Lady Frederick Cavendish opened the conference by appealing, as a Londoner deeply interested in the poor, to the country people to be very patient with the children when they showed evidences of London want of training. She reminded her hearers that they had been children themselves, and had probably not been quite devoid of the naughtinesses of childhood. Out of the 17,600 little Londoners who had been sent away under new influences it would be small wonder if some were occasionally troublesome. It must be remembered how bad was the discipline of the streets, which were generally the children's only playground in London. The children, moreover, when they got into the country and began to feel the benefit of fresh air and good, pure food, must be expected to get a fresh fund of almost wild spirits. They were often just saved from illness by the change of air. They gained immense pleasure from their new freedom, and every child-lover must feel their happiness a source of true pleasure. The Rev. J. Vallancey assured the company that in his village (Newport Pagnell) they regarded the children as a great blessing. Their occasional trespasses were easily condoned where general goodwill was felt. Among other stories, he related how a boy, who had stripped a tree of unripe currants, could not be persuaded that "those green things" could ever be of any use to anybody, and was indignant at the idea that he should be supposed to be so green as to imagine that they were the same article he saw in its ripe state in London. Mr. Vallancey gave some pleasant instances of the kindness of the country "mothers" to their temporary children, and said that an exchange of little presents during the ensuing winter was a common occurrence. Mr. Poulter then opened a discussion on the difficult question of sending children during their school holidays only. These are, unfortunately, only three or four weeks, and no organisation at present formed could arrange for the transit of 17,000 children in that time. Further, there are grave difficulties in the country, for naturally the cottagers prefer to have their spare beds filled as long as possible, and, indeed, many could not manage to keep any spare accommodation for merely a week or two, and the result might be overcrowding. On the other hand, Mr. Poulter, as a school manager, and after him two headmasters of large Board schools, dwelt with great emphasis on the very grave evils resulting from sending the children (unless so ailing as to require immediate change) in their school time. Regularity of attendance has to be most carefully fostered—regular children must not be taught irregularly, nor must the occasional attenders be encouraged to be absent. Further, one of the great moral benefits of the country holidays is the removal of the children during their idle time from the dangerous playground of the street. Again, the close co-operation and goodwill of the school teachers is most necessary for efficiently carrying out the selection of the most suitable cases. The Rev. W. P. Jay, a member of the London School Board, was appealed to as to the possibility of altering the times of the holidays, but the question has not yet apparently become prominent at the Board, however much the teachers and local managers may feel its importance. Mr. Jay dwelt on some of the difficulties of selection. It is very hard to please some of the country people—they expect to see the ragged and shoeless, and if the mother has pinched to make the child respectable, or the London Committee has given clothes, there is sometimes a suspicion that the really poor are not being sent. On the other hand, complaints are often made that the children have no "change of clothes," or that they are dirty in person or habits—a charge often, alas ! too true, but possibly a reason for greater care and kindness. Two or three country people spoke on minor points, and then the Hon. Dudley Fortescue, a member of the executive committee, proposed a vote of thanks to the Earl and Countess of Aberdeen for lending their house to the council. This was seconded by Bishop Selwyn, who spoke of the good done by a meeting in a West End square of so many persons thoroughly in touch with the very poor. He spoke feelingly on the division of classes, which struck anyone, who, like himself, came from the midst of a primitive society. He thought the miles of houses in the West End ought to be able to give every child of the poor a holiday.—A list of wants will be found in this week's *Nursing Mirror*.

